# Editorial: Emerging therapeutic approaches for non-alcoholic fatty liver disease

**DOI:** 10.3389/fmed.2024.1437385

**Published:** 2024-06-18

**Authors:** Mohamed El-Kassas, Khalid Alswat

**Affiliations:** ^1^Department of Endemic Medicine, Faculty of Medicine, Helwan University, Cairo, Egypt; ^2^Liver Disease Research Center, College of Medicine, King Saud University, Riyadh, Saudi Arabia; ^3^Steatotic Liver Disease Study Foundation in the Middle East and North Africa (SLMENA), Cairo, Egypt

**Keywords:** MASLD, MASH, NAFLD, treatment, non-invasive markers

Steatotic liver disease (SLD) exhibits a complex and multifaceted clinical spectrum, affecting a significant proportion of the world's population (1, 2). Consequently, the development pipeline for treatments targeting metabolic dysfunction-associated steatotic liver disease (MASLD), which was previously known as non-alcoholic fatty liver disease (NAFLD), and metabolic dysfunction-associated steatohepatitis (MASH), formerly known as non-alcoholic steatohepatitis (NASH), is expanding rapidly in several areas (3). Simultaneously, researchers are investigating various non-invasive tools for diagnosing and monitoring therapeutic interventions (4). Moreover, the applicability of these findings is crucial for optimizing outcomes in disease clinical trials (5). The recent introduction of new disease nomenclature and definitions has highlighted the importance of developing more precise and tailored treatment recommendations. Many of the pharmacotherapeutics in the MASLD/MASH pipeline have failed to obtain approval, while others, mainly used as monotherapies, have shown minimal benefits and are currently being investigated as components of combination therapies (6).

Nevertheless, some drugs are still being developed solely as individual treatments. Notably, Resmetirom has recently been approved by the Food and Drug Administration (FDA) as the first medical treatment for MASH (7). Other pharmaceutical options are under development, with the aim of specifically addressing MASH and fibrosis and reducing cardiometabolic risk factors (6). Additionally, repurposing current and approved medications is an attractive alternative due to the urgent need to develop new therapeutic strategies and the availability of cumulative safety and tolerability data. Recognizing MASLD as a multisystem disease is crucial, necessitating coordinated and interdisciplinary action plans (8). This Research Topic has collected several ground-breaking articles exploring various emerging therapeutic approaches to the disease. Machado reviewed the anticipated paradigm shift in managing MASLD, which resembles what occurred in hepatitis C management with directly acting antivirals a few years ago. The review summarized different approaches to managing MASLD, starting with lifestyle changes, which were long the sole treatment option, progressing through surgical and endoscopic bariatric interventions, and concluding with new pharmacotherapeutic agents. MASLD is always known to be the hepatic feature of adiposopathy, which also promotes other metabolic dysfunctions and increases the risk of cardiovascular diseases and cancers.

Machado emphasized the importance of targeting adiposopathy through weight reduction as an essential approach to managing NAFLD. Bariatric surgery in indicated patients, whether restrictive or combined restrictive and malabsorptive, has shown significant benefits in resolving steatosis and even regressing fibrosis in many patients. Endoscopic management of NAFLD, including intragastric/small intestinal devices and endoscopic sleeve gastroplasty, has advanced significantly in recent years.

Many antidiabetic drugs are being investigated for potential use in treating MASLD. Semaglutide, a human glucagon-like peptide-1 receptor agonist (GLP-1 RA), is a promising therapeutic option for treating patients with MASLD. Koureta and Cholongitas discussed the evolving role of semaglutide in NAFLD, highlighting its favorable effects on the components of metabolic syndrome, which is directly linked to NAFLD. The authors explored several studies investigating the impact of semaglutide in NAFLD that have shown improvements in liver steatosis. Nevertheless, the goal of regressing liver fibrosis remains challenging, and there is currently insufficient evidence in the literature to support the efficacy of semaglutide in reducing liver fibrosis sequelae. Hegazi et al. provided another perspective by reviewing clinical trials on herbal medications and dietary supplements for NAFLD based on the completed phase III and IV clinical trials shown on the ClinicalTrials.gov database. The search revealed a variety of nutraceuticals, with omega-3 fatty acids and vitamin D being the most investigated in NAFLD management. Addressing a crucial area in NAFLD management, Wang et al. discussed various potential diagnostic markers shared by NAFLD and atherosclerosis through machine learning and bioinformatic analysis. They applied machine learning algorithms to Gene Expression Omnibus (GEO) datasets to identify the most significant core genes for both conditions. They found 1,129 essential genes associated with NAFLD, 625 differentially expressed genes in atherosclerosis, and 47 genes common to both diseases. RPS6KA1 emerged as the most promising marker for diagnosing NAFLD and atherosclerosis, while SERPINA3 was identified as the most closely related gene for NASH and atherosclerosis.

In summary, MASLD is a worldwide health problem linked to metabolic dysfunction. This Research Topic offers a comprehensive overview of the recent updates on the management of MASLD. Given that MASLD is a multifaceted condition influenced by various risk factors such as genetic predisposition, environmental factors, and metabolic disorders, further well-designed clinical trials are still required to evaluate the possibility and efficacy of treating patients with MASLD by targeting their joint metabolic dysfunction to achieve the most effective treatment outcomes in the future.

## Author contributions

ME-K: Conceptualization, Data curation, Writing – original draft, Writing – review & editing. KA: Conceptualization, Data curation, Writing – original draft, Writing – review & editing.
